# Predictive model for coronavirus disease 2019 severity based on blood biomarkers: a retrospective study

**DOI:** 10.3389/fmed.2025.1597082

**Published:** 2025-08-08

**Authors:** Liu Xiaoyan, Bao Zhongying, Duan Shuhong, Sun Jing, Zhang Yijie, Zhang Jie, Liu Jingxin

**Affiliations:** ^1^Department of Infectious Diseases, Beijing Shijitan Hospital, Capital Medical University, Beijing, China; ^2^Department of Emergency, Beijing Shijitan Hospital, Capital Medical University, Beijing, China; ^3^Physical Education and Sports School, Soochow University, Suzhou, Jiangsu, China

**Keywords:** COVID-19, severity, blood biomarkers, predictive model, retrospective cohort analysis

## Abstract

**Objective:**

To develop and validate a clinical prediction model for assessing the severity of coronavirus disease 2019 (COVID-19) using blood biomarkers, aiming to support clinical decision-making and treatment guidance.

**Methods:**

A retrospective cohort study was conducted at Beijing Shijitan Hospital on January 5, 2023, including SARS-CoV-2 positive patients with initial chest CT-detected from outpatient and emergency departments. Data on demographics, symptoms, and blood biomarkers were collected. Patients were categorized into non-severe (mild and moderate) and severe (severe and critical) groups based on clinical symptoms and disease progression. Outpatient data served as the training set for modeling and validation using logistic regression and 10-fold cross validation. Emergency department data functioned as an independent external validation set to test the model’s generalizability.

**Results:**

The study included 1,007 patients, with 778 in the training set and 229 in the validation set. The C-reactive protein (CRP), neutrophil count (NE), neutrophil-to-lymphocyte ratio (NLR), platelet-to-lymphocyte ratio (PLR) were significantly higher in the severe COVID-19 group, while lymphocyte count (LY) and eosinophil count (EO) were significantly lower in the non-severe group (*p* < 0.001). The predictive model integrating these factors exhibited high discriminative power, achieving an AUC of 0.85, accuracy of 0.80, sensitivity of 0.73, and specificity of 0.81 in 10-fold cross validation, and an AUC of 0.86, accuracy of 0.82, sensitivity of 0.60, and specificity of 0.90 in the validation set.

**Conclusion:**

The predictive model, informed by blood biomarkers, successfully discriminates against COVID-19 patients at higher risk for severe outcomes, offering a valuable tool for clinical management and resource optimization.

## 1 Introduction

The coronavirus disease 2019 (COVID-19) pandemic, precipitated by the SARS-CoV-2 virus, emerged in 2019 and has rapidly disseminated across the globe, posing enormous challenges to public health (1–3). The mortality rate of COVID-19 was 0.91%, which is significantly lower than the mortality rates of SARS at approximately 10% and MERS at approximately 34% (2). Despite its lower mortality rate, COVID-19 has seen a succession of dominant strains, including Alpha, Beta, Gamma, Delta, and Omicron, each becoming the most prevalent and contributing to significant waves of infection (4, 5). Among these, Omicron is currently the global dominant strain, having rapidly outcompeted previous variants due to its high transmissibility and ability to evade immunity from prior infections or vaccinations (6). Although the Omicron variant has a lower severity and mortality rate, the large population, aging trend, and many high-risk individuals in China still pose significant challenges for managing the pandemic (7, 8).

Rapid identification of patients at risk for critical illness and early prognostic evaluation are essential for enhancing treatment efficiency. There were several tools for COVID-19 assessment, such as Sequential Organ Failure Assessment (SOFA), Acute Physiology and Chronic Health Evaluation II (APACHE II), and National Early Warning Score (NEWS). The SOFA score measures failure in respiratory, coagulation, hepatic, circulatory, neurological, and renal systems, while the APACHE II score combines the acute physiology score, an age component, and chronic health points, and the NEWS evaluates severity based on deviations in heart rate, respiratory rate, blood pressure, temperature, oxygen saturation, and level of consciousness. In a study of elderly patients with multiple comorbidities in the emergency department, NEWS demonstrated the highest predictive accuracy for COVID-19 severity with an AUC of 0.820, followed by APACHE II at 0.794 and SOFA at 0.784 (9). Despite this, these assessment tools are relatively complex and time-consuming in clinical applications, and some studies have begun to use a single immunological and inflammatory marker to predict the severity of COVID-19 (10, 11). Previous studies have found that C-reactive protein (CRP) has moderate predictive accuracy for severe or critical COVID-19, with an area under the curve (AUC) of 0.783 (12). Similarly, a retrospective cohort study of COVID-19 patients showed that the neutrophil-to-lymphocyte ratio (NLR) and platelet-to-lymphocyte ratio (PLR) also have moderate accuracy in assessing disease severity, with the AUC for NLR ranging from 0.59 to 0.81 and for PLR from 0.53 to 0.67 (13). This suggests that while these individual markers have some utility, their predictive power is limited when used alone.

Additionally, several studies have aimed to improve the accuracy of COVID-19 prediction models by combining multiple indicators. For instance, Araújo et al. (14) developed a machine-learning model that includes lymphocytes, mean corpuscular volume, platelets, red cell distribution width, and CRP, achieving an average AUC of 0.91 for predicting COVID-19 mortality within a 24-h window (14). Soares et al. (15) created a predictive model for COVID-19 severity with an AUC of 0.996 by integrating laboratory markers such as D-dimer, ferritin, neutrophil counts, haptoglobin, and soluble transferrin receptor, along with the metabolite cytosine, demonstrating high accuracy in identifying patients at risk for severe disease (15). However, these models have limitations. Machine learning models, despite their high AUC values for predicting mortality, are not practical in clinical settings due to their reliance on computer systems. Additionally, markers like D-dimer, ferritin, soluble transferrin receptor, and the metabolite cytosine are not routinely tested in outpatient settings.

Therefore, the aim of this study is to develop a model that can be used in clinical outpatient settings to efficiently and accurately predict COVID-19 disease severity, providing a useful clinical prediction tool and enhancing disease management.

## 2 Materials and methods

### 2.1 Study design

This study is a retrospective analysis of data collected from COVID-19 patients who visited the fever clinic and emergency department of Beijing Shijitan Hospital, affiliated with Capital Medical University, between 1 December 2022, and 31 January 2023. All patients tested positive for SARS-CoV-2 nucleic acid or COVID-19 antigen tests. During their outpatient visits, participants completed the recording of clinical symptoms and underwent testing for immunological and inflammatory markers, as well as high-resolution chest CT (HRCT) imaging.

Within two months post-admission, the hospital performed follow-up evaluations through the Health Information System (HIS) or telephone interviews to establish the definitive COVID-19 disease classification. Mild: Predominantly manifests as upper respiratory tract symptoms, including dry throat, sore throat, cough, and fever. Moderate: Defined by persistent high fever (> 3 days) and/or respiratory symptoms (e.g., cough, shortness of breath), accompanied by a resting respiratory rate (RR) < 30 breaths/min and oxygen saturation > 93% on room air, with imaging-confirmed COVID-19 pneumonia. Severe: Adults meeting ≥ 1 criterion (unrelated to other etiologies): (1) Shortness of breath with RR ≥ 30 breaths/min; (2) Resting oxygen saturation ≤ 93% on room air; (3) PaO2/FiO2 ratio ≤ 300 mmHg (1 mmHg = 0.133 kPa); (4) Radiographic evidence of > 50% lesion progression within 24–48 h. Critical: Patients fulfilling any criterion: (1) Respiratory failure requiring mechanical ventilation; (2) Shock; (3) Multiorgan failure necessitating ICU management.

The retrospective collection of clinical data from this phase began on 1 April 2024. This study was approved by the Ethics Committee of Beijing Shijitan Hospital affiliated with Capital Medical University and obtained a waiver of informed consent (IIT2024-008-002). In adherence to ethical standards, patient data have been anonymized to maintain confidentiality throughout the data processing phase.

#### 2.1.1 Inclusion criteria

Patients were included if they tested positive for SARS-CoV-2 nucleic acid or antigen tests and met the diagnostic criteria outlined in the “Diagnosis and Treatment Plan for COVID-19 (Trial Version 10th Edition)” issued by the National Health Commission of China in conjunction with the National Administration of Traditional Chinese Medicine on 5 January 2023.

#### 2.1.2 Exclusion criteria

Patients were excluded if they lacked comprehensive initial clinical data, blood tests, and lung CT scans; were unable to complete follow-up for disease and infection classification; had indistinct or ambiguous chest CT images; or had a history of hematological cancers.

### 2.2 Data collection

This retrospective study employed a standardized data collection protocol, with dual-researcher independent extraction of clinical and laboratory parameters. Initial clinical symptoms (cough, myalgia, sore throat, dyspnea, diarrhea, anorexia) were documented at presentation, while disease severity stratification was determined through electronic health record review or structured telephone follow-up 1–2 months post-visit. Hematological profiling was performed using the Mindray BC-5390-CRP automated analyzer to quantify C-reactive protein (CRP), leukocyte differential counts (WBC, neutrophils [NE], lymphocytes [LY], eosinophils [EO]), and platelet (PLT) levels, with derived inflammatory indices including neutrophil-to-lymphocyte ratio (NLR) and platelet-to-lymphocyte ratio (PLR). Thoracic imaging was acquired via a 32-detector Beijing Sinoway Insitum-CT338 scanner using standardized protocols: 120 kVp/150 mAs, 16 cm detector coverage, pitch 1.0, with 1.5 mm lung reconstructions (512 × 512 matrix, 380–450 mm FOV, lung window: WW1600/WL-600) and 5 mm mediastinal reconstructions (WW400/WL40), maintaining dose-length product ≤ 600 mGy⋅cm. Multiplanar reconstructions included coronal/sagittal views at 1 mm (lung) and 5 mm (mediastinal) increments. All CT studies underwent blinded independent analysis by two junior radiologists, with discordant interpretations resolved by senior radiologist adjudication. Quantitative imaging assessment focused on lesion topography (lobar distribution, axial/segmental localization), morphological characteristics (ground-glass opacity, consolidation patterns), and ancillary findings (pleural effusions, lymphadenopathy).

### 2.3 Statistical analysis

Patients were categorized into the non-severe/critical (non-SC) group and the severe/critical (SC) group based on the severity of their COVID-19 infection, with mild and moderate patients in the non-SC group, and severe and critical patients in the SC group. The categorical variables were presented as frequencies (%), and the chi-square test was used for intergroup comparisons. The normality of continuous variables was assessed. The data were reported as median (25th, 75th percentiles) for variables that were not normally distributed, and the Mann–Whitney U test was used for group comparisons. Conversely, normally distributed variables were described using mean ± standard deviation, with analysis of variance (ANOVA) employed for group comparisons. Variables showing significant differences were selected for model inclusion. To refine the diagnostic model variables, univariate logistic regression analysis was conducted to examine the association between clinical symptoms, blood markers, and disease severity. The variables for the multivariate logistic regression analysis were selected using the bidirectional stepwise regression method, and the outcomes of this analysis were used to develop nomograms for predicting disease severity risk.

In this study, data from the fever clinic were used as the training set to develop the model, with 10-fold cross validation applied to assess its internal validity. Meanwhile, data from the emergency department served as the test set to evaluate the model’s generalization ability. In the cross-validation process, StratifiedKFold was used to split the data into 10 folds while preserving the class distribution. SMOTE was applied to the training data of each fold to generate synthetic samples for the minority class, addressing class imbalance. The discriminatory power of these nomograms was evaluated through receiver operating characteristic (ROC) curves, which yielded area under the curve (AUC) values. These AUC values spanned from 0.5 to 1.0, with 0.5 representing the level of random chance and 1.0 indicating perfect accuracy. In addition to AUC, the performance of these models was further assessed using key metrics such as Accuracy, Sensitivity, Specificity, Positive Predictive Value (PPV), and Negative Predictive Value (NPV), with these calculations performed on both the training and validation set. Calibration curves were employed to assess the concordance between the predicted probabilities of COVID-19 severity classification and the actual severity of patients in both the training and validation sets. Calibration curves ensured the model’s reliability in classifying COVID-19 severity. Statistical significance was determined at a two-tailed *P*-value < 0.05. Data processing and analysis were conducted utilizing R 4.4.0, Python 3.8.20, and Zstats 1.0.

## 3 Results

### 3.1 Demographic and baseline characteristics of the patients in the training and validation sets

**FIGURE 1:**
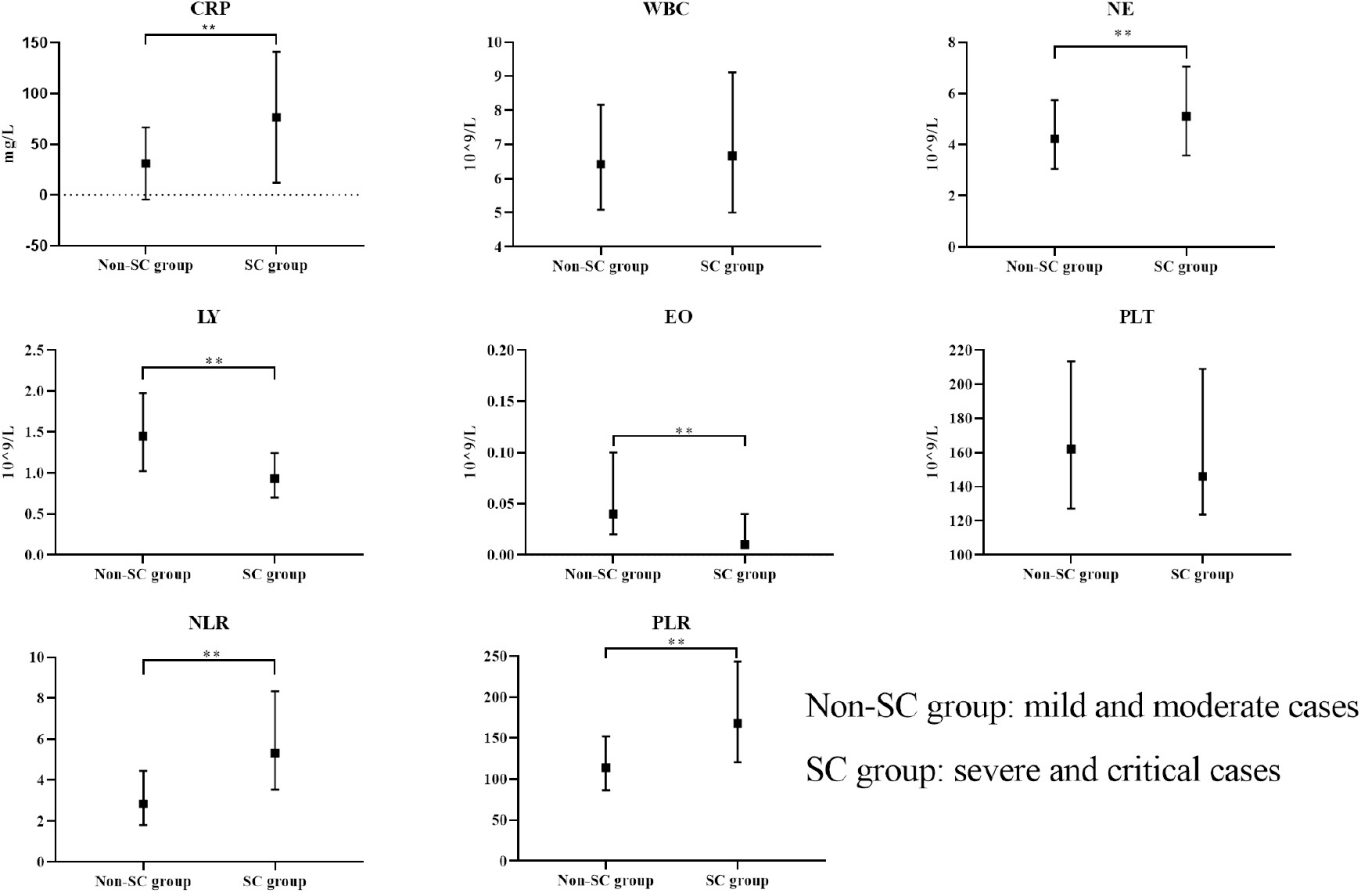
Comparison of immunological markers between non-SC and SC groups. The boxplots represent the median and interquartile range of each marker. **p < 0.01.

After excluding those who did not meet the criteria for COVID-19 and those with incomplete data or examinations, we included 778 participants. Among the 778 participants, 661 were classified into the non-SC group and 117 into the SC group, based on disease severity. The general information, clinical symptoms, immunological and inflammatory markers of the patients in the non-SC group and SC group were shown in Table 1. Participants in the SC group exhibited a higher prevalence of cough and sore throat compared to those in the non-SC group. Immunological and inflammatory marker analyses revealed that CRP, NE, NLR, and PLR levels were significantly elevated in the SC group compare to the non-SC group. In contrast, LY and EO levels were reduced in the SC group. These results imply that these markers may serve as potential indicators for classifying COVID-19 severity (Table 1 and Figure 1). The baseline characteristics of the patients in the validation sets are presented in the Supplementary Table S1.

**TABLE 1:** Baseline characteristics of the non-severe/critical (non-SC) group and the severe/critical (SC) group.

Variables	Non-SC group (*n* = 661)	SC group (*n* = 117)	Z/χ^2^	*P*-value
**Sex, *n* (%)**				
Male	355 (53.71)	70 (59.83)	1.50	0.220
Female	306 (46.29)	47 (40.17)		
Age, M (Q_1_, Q_3_)	65.00 (44.00, 76.00)	80.00 (69.00, 87.00)	−9.01	**< 0.001**
**Cough, *n* (%)**				
No	143 (21.63)	37 (31.62)	1.50	0.220
Yes	518 (78.37)	80 (68.38)		
**Muscle aches, *n* (%)**				
No	609 (92.13)	106 (90.60)	0.31	0.575
Yes	52 (7.87)	11 (9.40)		
**Sore throat, *n* (%)**				
No	356 (53.86)	78 (66.67)	6.61	**0.010**
Yes	305 (46.14)	39 (33.33)		
**SOB, *n* (%)**				
No	591 (89.41)	99 (84.62)	2.28	0.131
Yes	70 (10.59)	18 (15.38)		
**Diarrhea, *n* (%)**				
No	631 (95.46)	115 (98.29)	1.36	0.243
Yes	30 (4.54)	2 (1.71)		
**Loss of appetite, *n* (%)**				
No	641 (96.97)	114 (97.44)	0.001	1.000
Yes	20 (3.03)	3 (2.56)		
CRP, M [Q_1_, Q_3_]	19.45 (5.23, 41.77)	57.71 (29.40, 103.60)	−9.06	**< 0.001**
WBC, M [Q_1_, Q_3_]	6.42 (5.09, 8.16)	6.67 (5.01, 9.11)	−1.09	0.274
NE, M [Q_1_, Q_3_]	4.23 (3.05, 5.74)	5.11 (3.59, 7.04)	−4.05	**< 0.001**
LY, M [Q_1_, Q_3_]	1.45 (1.03, 1.97)	0.93 (0.71, 1.24)	−8.66	**< 0.001**
EO, M [Q_1_, Q_3_]	0.04 (0.02, 0.10)	0.01 (0.01, 0.04)	−6.24	**< 0.001**
PLT, M [Q_1_, Q_3_]	162.00 (127.00, 213.00)	146.00 (124.00, 207.00)	−1.13	0.261
NLR, M [Q_1_, Q_3_]	2.84 (1.82, 4.43)	5.31 (3.54, 8.32)	−9.10	**< 0.001**
PLR, M [Q_1_, Q_3_]	113.89 (86.46, 151.91)	168.18 (121.78, 242.35)	−7.30	**< 0.001**

The bold values indicate statistical significance. Z: Mann–Whitney test, χ^2^: chi-square test. M: Median, Q_1_: 1st Quartile, Q_3_: 3rd Quartile.

### 3.2 Identification of prognostic factors for disease severity

Variables without statistical significance in initial difference tests were still considered in univariate and multivariate logistic regression analyses to refine our variable selection process. This approach helps ensure that the final model possesses both stability and generalizability. Consequently, we performed univariate logistic regression on all variables to thoroughly evaluate our model for optimal stability and generalizability.

In the univariable logistic regression analysis, the reference values were determined as follows: for the gender variable, “Male” was used as the reference category, while for the symptom variables, the absence of each symptom was set as the reference. The univariate logistic regression analysis presented in Table 2 examines the relationships between various clinical symptoms, immunological and inflammatory markers, and COVID-19 disease severity.

**TABLE 2:** Univariate logistic regression analysis of blood indicators and the severity of COVID-19 disease.

Variables	β	S.E	Z	*P*	OR (95% CI)
Age	0.06	0.01	7.89	**< 0.001**	1.07 (1.05 ∼ 1.08)
**Gender**					
1					1.00 (reference)
2	−0.25	0.20	−1.22	0.221	0.78 (0.52 ∼ 1.16)
**Cough**					
0					1.00 (reference)
1	−0.52	0.22	−2.34	**0.019**	0.60 (0.39 ∼ 0.92)
**Muscle aches**					
0					1.00 (reference)
1	0.20	0.35	0.56	0.575	1.22 (0.61 ∼ 2.40)
**Sore throat**					
0					1.00 (reference)
1	−0.54	0.21	−2.55	**0.011**	0.58 (0.39 ∼ 0.88)
**SOB**					
0					1.00 (reference)
1	0.43	0.29	1.50	0.134	1.54 (0.88 ∼ 2.69)
**Diarrhea**					
0					1.00 (reference)
1	−1.01	0.74	−1.36	0.173	0.37 (0.09 ∼ 1.55)
**Loss of appetite**					
0					1.00 (reference)
1	−0.17	0.63	−0.27	0.786	0.84 (0.25 ∼ 2.88)
CRP	0.02	0.00	8.47	**< 0.001**	1.02 (1.01 ∼ 1.02)
WBC	0.09	0.03	2.73	**0.006**	1.09 (1.03 ∼ 1.17)
NE	0.17	0.03	5.02	**< 0.001**	1.19 (1.11 ∼ 1.28)
LY	−1.77	0.23	−7.67	**< 0.001**	0.17 (0.11 ∼ 0.27)
EO	−11.95	2.57	−4.65	**< 0.001**	0.00 (0.00 ∼ 0.00)
PLT	−0.00	0.00	−0.36	0.717	1.00 (1.00 ∼ 1.00)
NLR	0.14	0.02	6.67	**< 0.001**	1.15 (1.11 ∼ 1.20)
PLR	0.01	0.00	6.87	**< 0.001**	1.01 (1.01 ∼ 1.01)

The bold values indicate statistical significance. OR: odds ratio; CI: confidence interval.

Among these, significant predictors with *P*-values below 0.05 were CRP, WBC, NE, LY, EO, NLR, and PLR, which showed a statistically significant link to disease severity. Specifically, higher CRP, NE, and NLR were tied to greater odds of severe disease, as seen from their positive β coefficients and ORs over 1. Conversely, lower LY and EO levels were associated with reduced odds of severe disease, indicated by their negative β coefficients and ORs under 1 (Table 2).

### 3.3 Establishment of the nomogram prediction models and internal cross validation results

Utilizing the prognostic factors identified through multivariate logistic regression analysis in the training set (Table 3), each patient’s total score is calculated by summing the points assigned to variables such as age, cough, sore throat, CRP, WBC, LY, and EO. This cumulative score predicts the risk of disease severity, with detailed information provided in Table 3. The internal validation of the model was carried out via 10-fold cross validation. The results, presented in Table 4, show that the model has good performance, with an AUC of 0.85, accuracy of 0.80, sensitivity of 0.73, and specificity of 0.81 in the 10 validations.

**TABLE 3:** Multivariate logistic regression analysis of blood indicators and the severity of COVID-19 disease.

Variables	β	S.E	Z	*P*	OR (95% CI)
Intercept	−5.10	0.88	−5.77	**< 0.001**	0.01 (0.00 ∼ 0.03)
Age	0.06	0.01	6.24	**< 0.001**	1.06 (1.04 ∼ 1.08)
**Cough**					
0					1.00 (reference)
1	−0.65	0.30	−2.20	**0.028**	0.52 (0.29 ∼ 0.93)
**Sore throat**					
0					1.00 (reference)
1	−0.55	0.27	−2.09	**0.037**	0.57 (0.34 ∼ 0.97)
CRP	0.01	0.00	5.57	**< 0.001**	1.01 (1.01 ∼ 1.02)
WBC	0.10	0.04	2.36	**0.018**	1.11 (1.02 ∼ 1.20)
LY	−1.09	0.26	−4.22	**< 0.001**	0.34 (0.20 ∼ 0.56)
EO	−4.91	2.41	−2.04	**0.042**	0.01 (0.00 ∼ 0.83)

The bold values indicate statistical significance. OR: odds ratio; CI: confidence interval.

**TABLE 4:** Model validation and performance metrics for disease severity assessment.

	Internal validation (mean ± SD)	External validation (95% CI)
AUC	0.85 ± 0.08	0.86 (0.80–0.91)
Accuracy	0.80 ± 0.06	0.82 (0.76–0.86)
Sensitivity	0.73 ± 0.17	0.60 (0.48–0.72)
Specificity	0.81 ± 0.05	0.90 (0.86–0.95)
PPV	0.41 ± 0.09	0.71 (0.59–0.83)
NPV (95% CI)	0.95 ± 0.04	0.85 (0.80–0.90)

Internal validation was performed using 10-fold cross–validation, and external validation was conducted using an independent validation set. CI, confidence interval; AUC, the area under the curve.

### 3.4 External validation results of the nomogram prediction models

To further evaluate the model’s generalizability, it was applied to a new dataset. The external validation results, shown in Table 4, were similar to the internal validation results, with an AUC of 0.86, accuracy of 0.82, sensitivity of 0.60, and specificity of 0.90, indicating the model’s good generalizability (Table 4). In addition, we constructed a nomogram to enhance the model’s clinical applicability (Supplementary Figure S1) and plotted calibration curves for both the training (Supplementary Figure S2) and validation datasets (Supplementary Figure S3).

## 4 Discussion

The emergence of the COVID-19 pandemic, particularly the Omicron variant, underscores the critical importance of identifying risk individuals for severe outcomes to mitigate the burden on public health systems. Our study found that immunological and inflammatory markers demonstrated a stronger association with the risk of severe disease. Among these markers, we identified age, cough, sore throat, CRP, WBC, LY, EO as key variables for constructing a predictive model of COVID-19 disease severity. Following internal cross validation and external validation, the diagnostic model has demonstrated pronounced discriminative power and precise calibration capabilities, validating its potential for application in clinical practice for accurate diagnostic assessment of COVID-19 patients.

Patients with COVID-19 exhibit higher levels of circulating inflammatory cytokines and infection-related biomarkers, indicating a strong inflammatory response (16, 17). Among those biomarkers, reduced NE, and elevated in LY and NLR reflects a detrimental activation of the immune system in patients with COVID-19, was associated with severe disease and mortality in COVID-19 (18–21). Our study once again confirmed this phenomenon, finding that the LY and NLR in patients of the SC group were significantly higher than those in the non-SC group, while the NE was significantly lower than that in the non-SC group. Furthermore, some studies have also found that NLR and PLR can be used as rapid diagnostic tools to differentiate between mild and severe cases of COVID-19 (22). However, the accuracy of using NLR and PLR alone to predict disease severity is relatively low. In a retrospective COVID-19 cohort study, these ratios showed potential for predicting severity, with AUCs ranging from 0.59 to 0.81 for NLR and from 0.53 to 0.67 for PLR (13). Among the immunological and inflammatory markers, we should also pay attention to CRP. The level of CRP was elevated in the SC group compared to the non-SC group in our study, which was consistent with previous findings in COVID-19 studies (23, 24). The elevation of CRP levels reflects an exaggerated inflammatory response in COVID-19 patients. Previous study have found that the CRP was a key predictors of COVID-19 severity (25), the single CRP indicator can provide moderate predictive accuracy for severe or critical cases, with an AUC of 0.783 (12). Therefore, the diagnostic results of CRP once again indicate that a single indicator can provide moderate predictive accuracy, but it is not a perfect predictive tool. It may need to be used in conjunction with other biomarkers to improve the accuracy and reliability of predictions.

In our study, we identified a significant association between immunological and inflammatory markers and COVID-19 severity. Substantial deviations in these markers are indicative of potential severe disease progression. To rigorously evaluate the model, we employed 10-fold cross-validation coupled with external validation using independent cohorts, and the model demonstrated strong performance. It serves as an effective tool for personalized treatment and risk stratification by differentiating patients at high risk of severe disease progression. Additionally, we developed a nomogram to assess the risk of severe COVID-19. The model’s universality and clinical utility stem from its accurate prediction of disease severity using these common indicators, which is of critical importance for guiding clinical treatment. However, the following limitations should be considered. The study’s single-center sample may not reflect the diverse patient demographics and healthcare settings across different regions, and factors such as varied comorbidity profiles and distinct viral strains could potentially affect the model’s broader applicability and performance. Limited by study design and ethical constraints, the study was restricted to a single-center sample. Future studies can be designed with multi-center collaboration and ethical approval to enhance the model’s diagnostic accuracy.

## 5 Conclusion

In conclusion, the severity of COVID-19 is closely associated with age and specific immunological and inflammatory markers. Our study has established a predictive model for COVID-19 severity based on age, cough, sore throat, CRP, WBC, LY, EO demonstrating high accuracy in identifying high-risk patients and effectively guiding personalized treatment decisions. The model’s straightforward implementation process and cost-effectiveness make it a practical tool for clinical use. Overall, this study provides valuable insights for the assessment of COVID-19 patients, offering significant guidance for treatment strategies.

## Data Availability

The original contributions presented in this study are included in this article/Supplementary material, further inquiries can be directed to the corresponding authors.

## Supplementary material

Supplementary Figure S1 Nomogram for COVID-19 disease severity prediction.

Supplementary Figure S2 Calibration curve for the training set of the disease severity prediction model.

Supplementary Figure S3 Calibration curve for the validation set of the disease severity prediction model.
