# Supplementary figures and images for: Predictive model for coronavirus disease 2019 severity based on blood biomarkers: a retrospective study
Source: Front Med (Lausanne). 2025 Aug 8;12:1597082. doi: 10.3389/fmed.2025.1597082 (PMC12370689; doi:10.3389/fmed.2025.1597082)

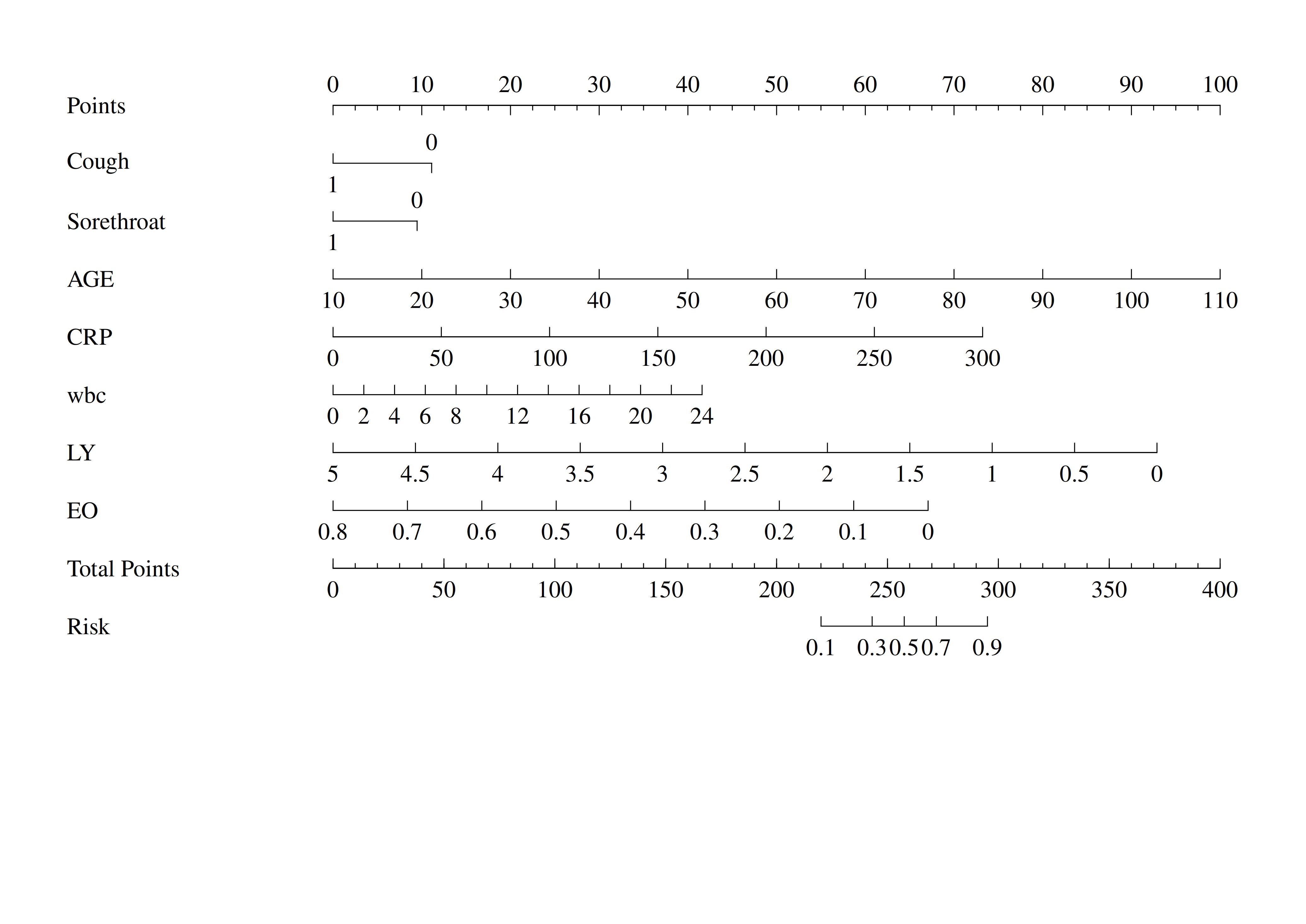

Supplement: Supplementary Figure S1 — Nomogram for COVID-19 disease severity prediction. [file Image_1.TIFF]

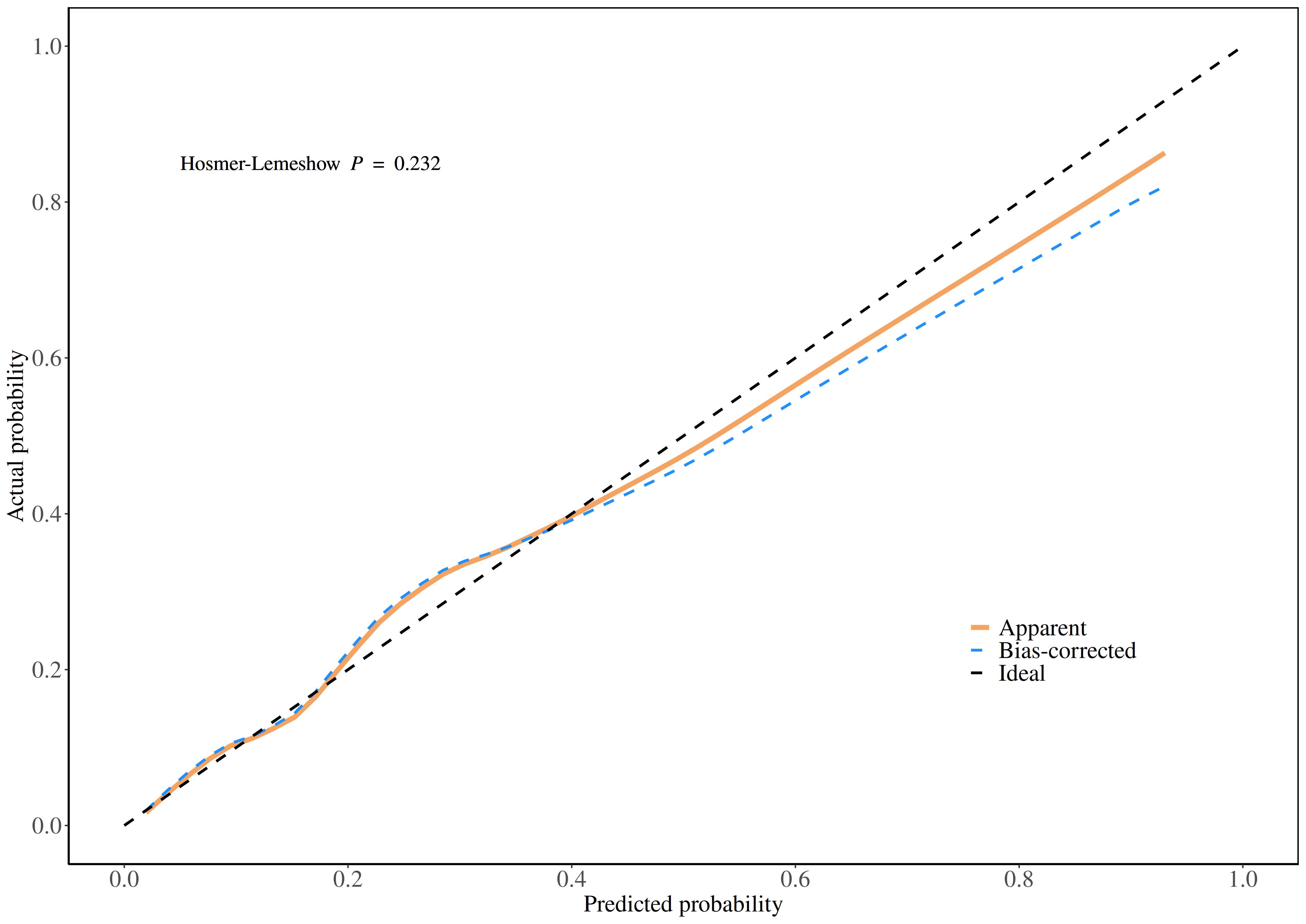

Supplement: Supplementary Figure S2 — Calibration curve for the training set of the disease severity prediction model. [file Image_2.TIFF]

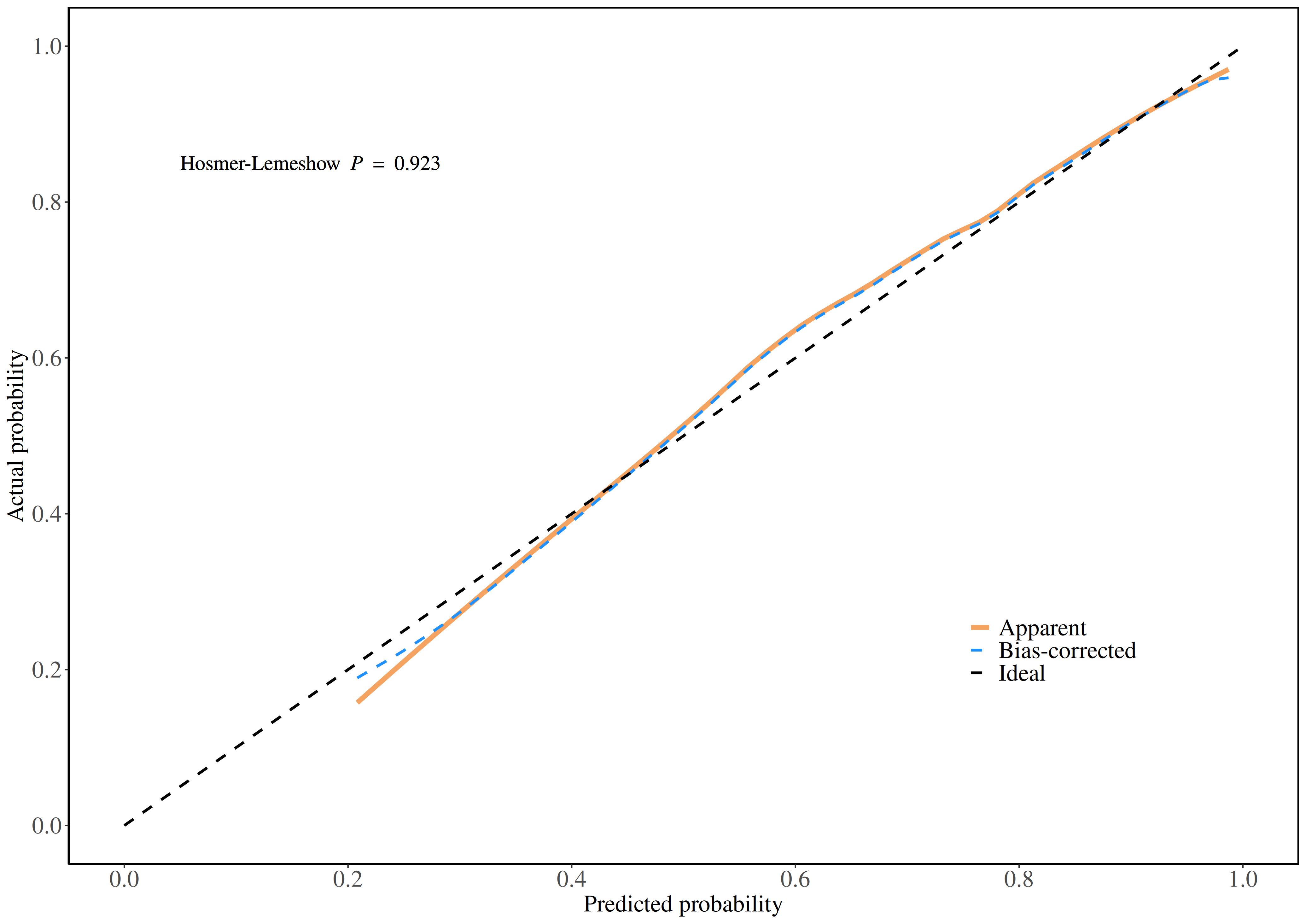

Supplement: Supplementary Figure S3 — Calibration curve for the validation set of the disease severity prediction model. [file Image_3.TIFF]
