# Supplementary material for: Predictive model for coronavirus disease 2019 severity based on blood biomarkers: a retrospective study
Source: Front Med (Lausanne). 2025 Aug 8;12:1597082. doi: 10.3389/fmed.2025.1597082 (PMC12370689; doi:10.3389/fmed.2025.1597082)
Supplement: Supplementary file 4 [file Table_1.docx]

Table S1 Comparison of baseline characteristics between non-sc and sc groups in the validation dataset

| Variables | Non-SC group (n=65) | SC group (n =164) | Z/χ² | *P* |
| --- | --- | --- | --- | --- |
|  |  |  |  |  |
| General Information and Clinical Symptoms | | | | |
| AGE, M (Q₁, Q₃) | 76.00 (63.00, 87.00) | 86.00 (79.00, 90.25) | -4.10 | **<0.001** |
| GENDER, n(%) |  |  |  |  |
| 1 | 34 (52.31) | 104 (63.41) | 2.40 | 0.121 |
| 2 | 31 (47.69) | 60 (36.59) |  |  |
| Cough, n(%) |  |  |  |  |
| 0 | 24 (36.92) | 45 (27.44) | 1.99 | 0.158 |
| 1 | 41 (63.08) | 119 (72.56) |  |  |
| Muscleaches, n(%) |  |  |  |  |
| 0 | 62 (95.38) | 160 (97.56) | 0.19 | 0.662 |
| 1 | 3 (4.62) | 4 (2.44) |  |  |
| Sorethroat, n(%) |  |  |  |  |
| 0 | 54 (83.08) | 155 (94.51) | 7.64 | **0.006** |
| 1 | 11 (16.92) | 9 (5.49) |  |  |
| SOB, n(%) |  |  |  |  |
| 0 | 44 (67.69) | 66 (40.24) | 14.05 | **<0.001** |
| 1 | 21 (32.31) | 98 (59.76) |  |  |
| Diarrhea, n(%) |  |  |  |  |
| 0 | 58 (89.23) | 147 (89.63) | 0.01 | 0.928 |
| 1 | 7 (10.77) | 17 (10.37) |  |  |
| Loss of appetite, n(%) |  |  |  |  |
| 0 | 58 (89.23) | 107 (65.24) | 13.30 | **<0.001** |
| 1 | 7 (10.77) | 57 (34.76) |  |  |
| Immunological and Inflammatory Markers | | | | |
| CRP, M (Q₁, Q₃) | 14.70 (5.85, 39.92) | 73.69 (34.48, 121.98) | -7.11 | **<0.001** |
| Wbc, M (Q₁, Q₃) | 6.24 (4.68, 8.38) | 7.76 (5.37, 11.31) | -3.62 | **<0.001** |
| NE, M (Q₁, Q₃) | 4.45 (3.11, 6.48) | 6.74 (4.02, 10.04) | -4.77 | **<0.001** |
| LY, M (Q₁, Q₃) | 0.91 (0.74, 1.47) | 0.64 (0.45, 0.90) | -5.13 | **<0.001** |
| EO, M (Q₁, Q₃) | 0.02 (0.00, 0.06) | 0.00 (0.00, 0.02) | -3.62 | **<0.001** |
| PLT, M (Q₁, Q₃) | 161.00 (129.00, 197.00) | 182.00 (130.75, 230.25) | -1.39 | 0.164 |
| NLR, M (Q₁, Q₃) | 4.16 (2.35, 7.28) | 10.33 (6.69, 17.13) | -6.75 | **<0.001** |
| PLR, M (Q₁, Q₃) | 175.82 (109.94, 227.03) | 280.49 (181.28, 419.65) | -5.08 | **<0.001** |
